# Facts and Observations in Medicine and Surgery

**Published:** 1845-01

**Authors:** 


Art. XI.—*Facts and Observations in Medicine and Surgery; the gleanings of ten years of active general practice, and having particular reference to Fractures and Dislocations, Gunshot Wound* [*s*], *Calculus, Insanity, Epilepsy, Hydrocephalus, the Therapeutic application of Galvanism, and Fibrinous Diarrhea.* By John Grantham, &c. &c.—*London*, [*no date.*] 8vo, pp. 216.

A book sometimes fulfils, sometimes fails to fulfil, the promise of its title-page. The work before us, we are sorry to say, must be classed among the latter. The notices, somewhat ostentatiously paraded, of "Fractures and Dislocations, Gunshot-wound, Calculus," &c., appear, on inspection, to contain nothing particularly requiring or warranting their transference from the 'Medical Gazette,' where, we are informed in the preface, they originally appeared, and their reproduction in a volume. We do not deny that several of the articles are respectable contributions to periodical literature, and were probably useful in their original sphere; but what we object to is, the needless addition of an octavo volume holding forth hopes which it by no means realizes, to our already cumbrously large collection of medical literature.

Of the two parts into which the work is divided, the surgical is the most worthy of attention, yet, even in this, with one or two exceptions, there are no facts or cases of a novel or particularly interesting character.

One case, entitled "Granulating surface, measuring 600 square inches, recovery," contains the history of a boy, who was extensively scorched, by an explosion of fireworks, and in whom, the upper and fore part of the neck, the left arm to the insertion of the deltoid, both axillæ, and round the posterior part of the body, to within three inches of the spines of the vertebræ, the chest, body, and genitals, to the verge of the anus, the upper part of the right thigh and the left thigh to the knee, were denuded of cuticle, rete mucosum, and corium, as well as of the subcutaneous structure, so that the "arteries and veins were seen, as if neatly dissected, lying on the surface of the muscles and fascia. (p. 91.) Mr. Grantham's treatment of this case, as of several others recorded in his book, seems to have been extremely judicious and very creditable to him, although, at the same time, nothing in that treatment was new or remarkable.

At page 100, a case of rupture of the rectus femoris is given. It was that of "a stout, hale man," who was running with his son. Mr. Grantham did not see the man till six weeks after the accident had occurred, when he found the muscle "drawn one third up the thigh, and with a corresponding depression above the patella." (p. 101.) Of course, nothing could be done: but Mr. Grantham informed the patient, the "power was gone, but that it would be supplied from the two vasti muscles." (p. 102.)

In conclusion we would say, that while the contents of this volume prove Mr. Grantham to be as ableand judicious a practitioner as we know him to be a most respectable man, we are still of opinion that he would have best consulted his own reputation by leaving them in their original locality.

We must also complain of the style of this work. It is perhaps not easy to speak with too great severity of its incorrectness.

Formerly, a grammatical knowledge of language and a correct style were thought essential in a writer of books; but many of our medical authors do not seem to trouble themselves about such matters. But we would have them to know, that a man has no more business to *write* until he has mastered his language, than he has to *practise* until he has mastered his physic and his surgery.

